# Regulation of microRNAs in Satellite Cell Renewal, Muscle Function, Sarcopenia and the Role of Exercise

**DOI:** 10.3390/ijms21186732

**Published:** 2020-09-14

**Authors:** Stefania Fochi, Gaia Giuriato, Tonia De Simone, Macarena Gomez-Lira, Stefano Tamburin, Lidia Del Piccolo, Federico Schena, Massimo Venturelli, Maria Grazia Romanelli

**Affiliations:** 1 Department of Neurosciences, Biomedicine and Movement Sciences, University of Verona, 37134 Verona, Italy; stefania.fochi@univr.it (S.F.); gaia.giuriato@univr.it (G.G.); tonia.desimone@univr.it (T.D.S.); macarena.gomezlira@univr.it (M.G.-L.); stefano.tamburin@univr.it (S.T.); lidia.delpiccolo@univr.it (L.D.P.); federico.schena@univr.it (F.S.); massimo.venturelli@univr.it (M.V.); 2 Department of Internal Medicine, University of Utah, Salt Lake City, UT 84132, USA

**Keywords:** microRNA, aging, sarcopenia, myogenesis, exercise, satellite cells

## Abstract

Sarcopenia refers to a condition of progressive loss of skeletal muscle mass and function associated with a higher risk of falls and fractures in older adults. Musculoskeletal aging leads to reduced muscle mass and strength, affecting the quality of life in elderly people. In recent years, several studies contributed to improve the knowledge of the pathophysiological alterations that lead to skeletal muscle dysfunction; however, the molecular mechanisms underlying sarcopenia are still not fully understood. Muscle development and homeostasis require a fine gene expression modulation by mechanisms in which microRNAs (miRNAs) play a crucial role. miRNAs modulate key steps of skeletal myogenesis including satellite cells renewal, skeletal muscle plasticity, and regeneration. Here, we provide an overview of the general aspects of muscle regeneration and miRNAs role in skeletal mass homeostasis and plasticity with a special interest in their expression in sarcopenia and skeletal muscle adaptation to exercise in the elderly.

## 1. Introduction

Sarcopenia is a pathophysiological process characterized by progressive loss of skeletal muscle mass and function that contributes to a higher risk of falls and fractures among older adults [1]. It often leads to frailty that impacts considerably on the quality of life in elderly people. After the age of 50, the rate of muscle loss is estimated to be 1–2% per year, being faster in men than women [2]. The advances in transcriptomics technologies and high throughput analyses allow for detection of a broad repertoire of cellular factors that participate to gene expression regulation during skeletal muscle aging changes and adaptations such as growth factors, transcription factors, cell signaling pathways activators, and non-coding RNA, including microRNAs (miRNAs) [3].

miRNAs are a class of non-coding transcripts specifically involved in negatively modulated gene expression at the post-transcriptional levels by specifically targeting the 3′-untranslated regions (3′-UTR) of their target mRNAs. Several miRNAs have been demonstrated to participate in the molecular mechanisms that control skeletal muscle plasticity during aging. miRNAs that are preferentially expressed in striated muscle are termed as myomiRs, and they act as regulators of muscle development, homeostasis, and functionality [4,5]. Expression of skeletal myomiRs may be altered in old skeletal muscle, in age- and activity-related muscle changes (e.g., hypertrophy, atrophy), as well as in myopathies, such as muscular dystrophy [6,7,8,9]. Recent studies have demonstrated that muscle-specific miRNAs play a pivotal role in the control of sarcopenia, modulating key steps of skeletal myogenesis, including satellite cells senescence, cell proliferation, and differentiation [10,11].

The reduction in force of skeletal muscle in older people is primarily due to the loss and weakening of muscle fibers. For instance, during aging, the locomotor skeletal muscles face a marked switch to slow type I myofibers, a process of atrophy of fast (type IIa and IIx) fibers, with a reduction in the myofiber count [12,13,14]. Similar changes have been associated with sedentary lifestyles, and reported in some neuromuscular disorders [6,15,16,17,18]. Thus, exercise is considered an effective intervention to delay the onset and progression of sarcopenia. Regular exercise training has been associated with an increase in skeletal muscle cross-sectional area, force, and resistance to fatigue [19], as well as improvements in cardiovascular fitness and quality of life [20]. Exercise training promotes positive adaptations in skeletal muscle, acting as an important stimulator of extra/intracellular signals, leading to changes in gene expression. Several studies have revealed that miRNA expression in skeletal muscle is modulated in response to physical exercise, see the exhaustive reviews [21,22,23], highlighting the relevance of the miRNAs role in the process of adaptation to exercise. More recently, circulating miRNAs have been investigated as possible biomarkers of exercise training [24].

This review will synthesize the most relevant advancements in the knowledge of the miRNAs role in muscle regeneration and their involvement in sarcopenia. We will further review the current knowledge on circulating miRNAs and their potential as non-invasive biomarkers of muscle function and adaptation to exercise in healthy adults and sarcopenic elderlies.

## 2. miRNA Biogenesis and Functional Role

miRNAs are small non-coding RNAs of 20 nucleotides in length, transcribed by about 2000 human genes, that regulate gene expression at the post-transcriptional level [25,26]. miRNAs play crucial roles in various biological processes such as cell proliferation, apoptosis, differentiation as well as epigenetic changes and metabolic homeostasis. miRNA genes are often found in clusters and may derive from intragenic or intergenic regions, both intronic and exonic [27]. miRNAs biogenesis begins with the synthesis of primary miRNAs (pri-miRNAs) by RNA polymerase II that are processed in the nucleus into precursor miRNAs of approximately 70 nt (pre-miRNAs) by a complex consisting of the RNA binding protein, DGCR8 microprocessor subunit, and the endoribonuclease, Drosha [28]. Pre-miRNAs are then exported to the cytoplasm via the nuclear transport protein Exportin 5, where the endoribonuclease Dicer cuts the stem loop region producing miRNAs duplexes. miRNAs duplexes harbor the mature miRNA strand that, once associated with the RNA induced silencing (RISC) complex, works as a gene modulator at the transcriptional level. 

miRNAs regulate transcripts expression generally by annealing to the 3′ untranslated region (3′UTR), leading to inhibition of mRNA translation and/or degradation of mRNA transcripts. miRNA may target mRNA not only at their 3′-UTR, but also at the 5′UTR, promoters and exon sequences, generally leading to downregulation of the expressed genes [29]. Each miRNA can target several mRNAs involved in different cell functions and in a crosstalk between cell signaling pathways. Deregulation of miRNA expression has been associated with several pathological processes, including cancer, neurodegenerative diseases, cardiovascular diseases, and host response to viral infections [30,31,32,33].

## 3. Skeletal Myogenesis and Sarcopenia

**Figure 1:**
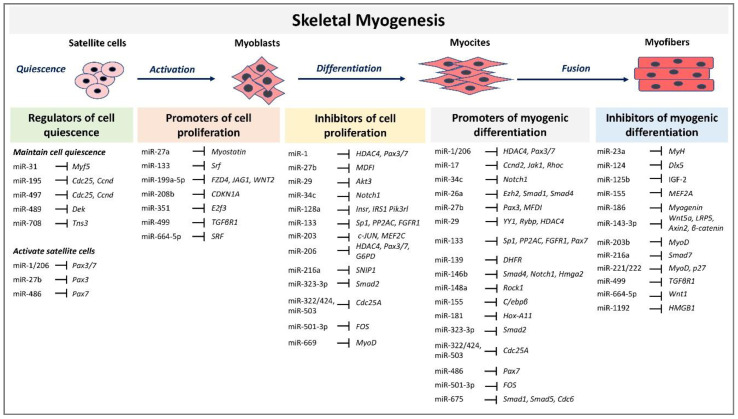
MicroRNAs (miRNAs) and target genes implicated in skeletal muscle myogenesis.

Skeletal myogenesis refers to the process of muscle development, whereby myogenic precursor cells can differentiate and fuse to form myofibers. During development, most of the precursor cells undergo proliferation and differentiation, while a subset remains capable of regeneration, repairing tissue injuries in adulthood. Myogenesis involves an intricate regulatory network of gene expression that coordinates (i) activation and proliferation of stem cells that differentiate into myoblasts, (ii) early differentiation that consists in the fusion of myoblasts to form myocytes and (iii) terminal differentiation into myofibers (Figure 1). The skeletal myogenic process is finely regulated by transcription factors known as myogenic regulatory factors (MRFs), such as MyoD (myogenic differentiation), myogenin, Myf5 (myogenic factor 5), and MRF4 (myogenic regulatory factor 4) [34]. In adults, muscle stem cells, termed satellite cells, are characterized by the expression of the paired box transcription factor Pax7 and to a small extent of Pax3, whereas MyoD is not expressed. This pattern of expression allows the maintenance of satellite cells in a quiescent status. Activation of satellite cells from the quiescent status is first determined by a co-expression of Pax7, MyoD, and Myf5 [3]. At this step, Pax factors promote the expression of genes accountable for inducing a transient cell proliferation suppressing genes that induce differentiation. During myogenic differentiation, the expression of Pax proteins is suppressed, allowing the expression of MRFs. At this step, myogenin plays a key role in the terminal differentiation of myogenic progenitor cells, whereas MyoD undergoes to downregulation. In the aging muscle, the expression of the myogenic genes, MyoD, and myogenin, have been reported to be upregulated, suggesting a modification of the committed status of the muscle satellite cells [35].

The altered ability of satellite cells to maintain quiescent status and their loss of self-renewal and regenerative capacity are major causes of age-related muscle changes [36]. In skeletal muscle, aging is manifested as sarcopenia, the progressive loss of skeletal muscle mass and strength with accumulation of functional deficits [17,18,37]. This age-related muscle atrophy strictly correlates with physical disability, poor quality of life and increased risk of negative outcomes for elderly people. From a cellular point of view, satellite cell depletion, senescence, inflammation, and mitochondrial dysfunction have been proposed to be associated with sarcopenia development [38,39,40,41].

A decrease in the number of stem cells and in their capability to proliferate during aging has been proposed to impact myogenesis [42]. Study in mice have demonstrated that reduction of satellite cells affects muscle regenerative capacity without affecting sarcopenia, and does not contribute to the maintenance of muscle size during aging, however in sedentary adult mice, in the absence of injury, satellite cells may contribute to myfibers at different extent between muscle and with age [43,44]. More recently, it has been also demonstrated that satellite cells depletion in adult mice contributes to preserve physical function and to increase in muscle fiber size when physical activity is lifelong sustained [45]. In humans, the role of satellite cells in contributing to hypertrophy and to regeneration in the elderly is not clearly defined, also due to the difficulty of studying muscle regeneration in human subjects. Satellite cells may be responsible for reduced regenerative muscle potential in older individuals due to the switching of reversible quiescence into senescence [40]. Interestingly, a recent study has contributed to better understanding the role of satellite cells in elderly individuals compared to young ones, in a regeneration model obtained inducing muscle injury by electrically stimulated eccentric contraction of the vastus lateralis, followed by 13 weeks of resistance training. Surprisingly, the results clearly demonstrate that satellite cells increase in young and elderly to a similar extent, confirming a complete muscle regeneration, whereas the increased number of satellite cells does not enhance hypertrophy in response to resistance training [46].

Recently, attention has been directed to the role of inflammation in the development of sarcopenia. Increased inflammation in the elderly, the so-called inflammaging, is driven by the expression of proinflammatory cytokines activated by transcription factor nuclear factor-kappa B (NF-κB) and response to oxidative stress and has been considered a crucial mediator of muscle wasting in sarcopenia [47,48,49]. Increasing evidence shows that mitochondrial dysfunction is a cause of sarcopenia, contributing to inflammation via reactive oxygen species (ROS) production and NF-κB activation, accelerating the aging process of skeletal muscle [50]. The decline of mitochondrial function is a typical feature of the senescence-associated secretory phenotype of senescent cells, which leads to muscle wasting [51]. It is not clear if during aging muscle cells undergo an increase of cell senescence that may contribute to sarcopenia. However, a recent study had attempted to quantify senescent cells in human skeletal muscle using the phosphorylated form of the histone H2A variant gamma-H2AH (γH2AX) as a marker of DNA damage. In muscle cell biopsy of young subjects compared to elderly ones, the latter did not show a significant increase of senescent cells [52]. Interestingly, the same study demonstrated a higher cells senescence and senescence-associated secretory phenotype in muscle from obese individuals and in in vitro obesogenic environments, suggesting that DNA damage, which may reduce the function of the muscle, is more directly linked to obesity than aging.

## 4. The Role of miRNAs during Skeletal Myogenesis in Adults

MRFs expression can be regulated by miRNAs highlighting their involvement in modulating myogenesis. A set of miRNAs, referred to as myomiRs, such as miR-1, miR-133a, miR-133b, miR-206, miR-208, miR-208b, miR-486, and miR-499, are highly enriched in muscle fibers [4]. MyomiRs may modulate size and type transition of muscle fibers in structural adaptation to aging and exercise. Specifically, miR-499, miR-208 and miR-208b, encoded by intron sequences of myosin heavy chain genes, have been demonstrated in mice models to participate in a network of miRNAs that regulate not only myosin expression, but also fiber type genes expression and muscle performance [53]. Skeletal muscle-specific Dicer knockout in mice results in low expression of specific miRNAs, reduced skeletal muscle mass, and abnormal myofibers morphology, confirming that a correct maturation of miRNAs is necessary for muscle development and function [54]. In adult mice, depletion of Dicer has been demonstrated to reduce the expression of miR-1, miR-133a and miR-206 expression; however, this reduction had no effect on skeletal muscle mass and phenotype, even after a long-life reduction [55]. In contrast, the reduction of myomiRs after Dicer knockout affects skeletal muscle regeneration [56]. Unexpected results derived by Dicer depletion studies in adult mice skeletal muscle showed that, even if Dicer expression might be reduced of almost 80%, myomiRs expression was not as much significantly reduced. This finding opens interest for further studies that may investigate the effectiveness of Dicer function and myomiRs stability and function in adult muscle. Several reviews have extensively described the role of miRNAs during embryonal skeletal myogenesis [8,9,57]. Here we report and discuss the most recent evidence concerning miRNA regulatory network in adults. A selected list of myomiRs and miRNAs implicated in the different phases of skeletal myogenesis from quiescence to the proliferation of satellite cells and myofibers differentiation are represented in Figure 1.

### 4.1. miRNAs Role in the Control of Satellite Cells Quiescence Status

During the first steps of myogenesis or muscle regeneration, miRNAs can affect the expression of MRFs, Pax3/7 transcription factors and other transcripts that control the cell cycle, playing a key role in the maintenance of cell quiescence status or in the activation of satellite cells [4,58]. In satellite cells of adult mice, the ablation of Dicer results in the interruption of quiescence status and impaired ability of injured muscle regeneration [59]. The essential role of miRNAs in maintaining stem cells proliferation is confirmed by the observation that Dicer deletion in adult mice muscle cells causes apoptosis of proliferating satellite cells [59,60].

Several miRNAs have been found to play a crucial role in the maintenance of satellite cells in a quiescence status. One of the miRNAs most intensively studied is miR-31, which has been demonstrated to directly participate in the regulation of myogenic transcription factors. miR-31 has been reported to downregulate the expression of Myf5 in quiescent satellite cells by sequestering Myf5 in messenger ribonucleoprotein particles (mRNPs) [61]. Activation of satellite cells allows mRNPs dissociation and the release of Myf5 transcripts that are rapidly translated, promoting the myogenesis process. Another mechanism by which miRNAs control satellite cells quiescence is by targeting the cell cycle regulators. Sato and co-workers demonstrated a close link between quiescence and inhibition of myogenesis in adult satellite cells. miR-195/497 convert the proliferating juvenile muscle satellite cells into quiescent ones directly targeting cell division cycle 25C (Cdc25) and cyclin D (Ccnd) factors [62]. Conversely, inhibition of miR-195/497 results in the activation of Cdc25 and Ccnd and reduction of Pax7 expression. Although in silico analyses show no evidence for direct targeting of Pax7 or MyoD transcripts by miR-195/497, their action on Cdc25/Ccnd transcripts elicit the upregulation of Pax7 and downregulation of MyoD expression, which are characteristics of quiescent adult skeletal muscle cells [62].

Proliferative expansion of myogenic progenitors is regulated by another miRNA, miR-489, which has been demonstrated to inhibit the expression of the oncogene Dek that regulates cell proliferation and mRNA splicing. miR-489 is highly expressed in quiescent satellite cells and is downregulated during their activation. Induced expression of Dek during asymmetric division of satellite cells promotes the transient proliferative expansion and differentiation of myogenic progenitors, whereas Dek is absent in self-renewing cells [60].

A new regulatory axis that controls the satellite quiescent cells transition to their activated state involving Notch, miR-708 and Tensin 3 (Tns3) has been recently proposed [63]. Notch signaling is known to stimulate the proliferation and to inhibit the differentiation of muscle satellite cells. Notch-mediated induction of miR-708 transcription allows the inhibition of Tns3 transcript, a regulator of cell migration. Low levels of Tns3 mediated by miR-708 allow the anchored quiescent status of satellite cells. Upon activation of satellite cells, Notch and therefore miR-708 are downregulated, resulting in high levels of Tns3 and the migration of activated cells, suggesting that suppression of the migratory ability is essential for maintaining the quiescent status of stem cell within its niche, where miR-708 acts as a gate-keeper of quiescence [63].

### 4.2. miRNAs Role in Myogenesis

Once satellite cells are activated, they first undergo a rapid and short step of cell proliferation followed by a phase of cell differentiation. In skeletal muscle, the processes of proliferation and differentiation are mutually exclusive. Indeed, various miRNAs here described as promoters of cell differentiation, are also inhibitors of cell proliferation and vice versa. Several miRNAs participate to satellite cells myogenic differentiation mainly targeting transcripts encoding transcription factors, cell cycle regulators and cell signaling pathways factors. miR-1/206 family, miR-27b, miR-486 and miR-133b have been demonstrated to downregulate the expression of the transcription factors Pax3/7 allowing satellite cell activation [64,65,66,67,68,69]. The reduction of Pax7 protein levels is mediated by miR-1 and miR-206 in activated satellite cells. Overexpression of miR-1 and miR-206 has been demonstrated to restrict the proliferative potential of satellite cells and to accelerate their myogenic differentiation [65]. miR-1 and miR-206 have also been reported to target Pax3, which leads to the timely expression of myogenin expression in committed myoblasts [68]. A model of miR-1/206 function that requires an initial step of Pax3-mediated MRFs activation to induce the expression of miR-1/206, which in turn repress Pax3 in a negative feedback loop, has been proposed [68]. A recent study identified the glucose-6-phosphate dehydrogenase (G6PD) gene as a new target of miR-206 [70]. Suppression of G6PD by miR-206 results in the inhibition of muscle cell proliferation and cell cycle arrest in G0/G1 phase. In addition to miR-1/206, miR-27b, and miR-486 have been also demonstrated to promote satellite cells differentiation downregulating Pax proteins expression. Overexpression of miR-27b results in a specific downregulation of Pax3, not effecting Pax7 expression. In vivo analyses revealed that injection of miR-27b antagomirs at a site of muscle injury alters levels of Pax3 and affects the regeneration of injured muscle [64], suggesting the possibility to modulate the Pax gene expression by miRNA injection as a new challenge in the development of therapeutic strategies against muscle damage.

In an additional study, Dey and colleagues propose an intricate regulatory network between Pax7, miR-486 and miR-206 [67]. Pax7 expression in the satellite cells differentiation step mediates the activation of the inhibitor of DNA Binding 2 (Id2) factor, which suppresses MyoD-mediated transcriptional activation of miR-486 an miR-206. Conversely, in a MyoD-dominated state, the upregulation of miR-486 and miR-206 allows repression of Pax7 and Id2, allowing a shift of the equilibrium toward the MyoD-active myotube state.

Both miR-1 and miR-133 have been associated with skeletal myogenic processes. miR-1 and miR-133 are clustered on the same chromosomal loci (miR-1-1 and miR-133a-2 on chromosome 20, miR-1-2 and miR-133a-1 on chromosome 18) and transcribed together as a single transcript, becoming then two mature miRNAs that suppress different target genes. An early study revealed that increased levels of miR-1 promote myogenesis by suppressing the expression of histone deacetylase 4 (HDAC4) [71]. HDAC4 has been shown to inhibit muscle differentiation, mainly reducing myocyte enhancer factor 2 (MEF2), suggesting that miR-1 mediated suppression of HDAC4 may promote cell differentiation. If the miR-1 activity in promoting cell differentiation is commonly accepted, the effect of miR-133 activity in the skeletal myogenic process is more debated. At first, it has been demonstrated that miR-133a suppresses the expression of serum response factor (SRF), a central regulator of muscle cells proliferation and differentiation, enhancing myoblast proliferation. Further studies suggest that miR-133 may reduce myoblast proliferation, inducing myoblasts cell differentiation directly targeting the transcription factor Sp1 [72]. In addition, miR-133 may suppress extracellular signal-regulated kinases ERK1/2 expression, through targeting FGFR1 and PP2AC, which are part of a pro-proliferation signaling cascade [73]. Recently, it has been demonstrated that Wnt3, a member of the Wnt signaling, well-known to promote myoblast differentiation, increases the expression of miR-133b and miR-206, but does not influence the expression of miR-1 and miR-133a [69]. miR-133b targets Pax7, suppressing its expression more efficiently than miR-206 by targeting a site adjacent to the miR-206 binding site in the Pax7 3′UTR [69].

Other miRNAs have been implicated in the promotion of myogenic differentiation targeting specific cell signaling pathways and cell cycle regulators. Early studies revealed that the upregulation of two specific miRNAs, miR-26a and miR-214 during myogenesis correlates with a decline in the expression of enhancer of zeste homolog 2 (Ezh2), a chromatin-modifying enzyme that negatively regulates myogenesis [74,75]. miR-26a has also been found to be upregulated in both mice and human skeletal muscles and it has been demonstrated to inhibit transforming growth factor-beta (TGFβ) signaling by targeting Smad1 and Smad4 [76]. Alteration of TGFβ signaling pathway, whose members are potent inhibitors of myoblasts differentiation mainly affecting the expression of MyoD and myogenin, is a strategy adopted by various miRNAs to regulate myogenesis. Furthermore, miR-675 has been shown to target Smad1, Smad5, and cell division cycle 6 (Cdc6) promoting cell differentiation [77].

Specific miRNAs have also been demonstrated to promote myogenesis by suppressing the expression of various targets belonging to the inflammatory NF-κB signaling pathway. It has been demonstrated that miR-29 targets YY1 (yin yang 1), a transcription factor downstream of the NF-κB signaling and YY1 binding protein (Rybp), participating to a feedback loop mechanism that ensure myogenic differentiation. YY1 and Rybp act together to impair myogenesis by suppressing the expression of myogenic genes. They form a repressive complex Rybp/YY1/Ezh2/HDAC4 that epigenetically inhibits miR-29. Upon myogenesis, MyoD/SRF displace this repressive complex promoting miR-29 expression and myogenic differentiation [78,79].

miR-322/424 and -503 have been demonstrated to repress the expression of cell division cycle 25A (Cdc25A) protein phosphatase during myoblast C2C12 differentiation [80].

More recently, miR-17 and miR-19 have been demonstrated to act in concert to promote cell differentiation and muscle regeneration after injury in vivo [81]. miR-17 mediates repression of regulators of cell proliferation, such as the cyclin Ccnd2 and the tyrosine kinase protein Jak1, and regulators of cell fusion and repressors of cell motility, such as the small G protein Rhoc. Furthermore, the miR-17 expression has been shown to correlate to the upregulation of the transcription factors Myh3 and MyoD1 [81].

An additional regulatory feedback loop involving miRNAs in promoting satellite cells differentiation has been demonstrated to reduce MyoD family inhibitor (MDFI) expression. miR-27b has been shown to inhibit the proliferation of pig satellite cells and to promote their differentiation in vitro, through the suppression of MDFI [82]. MDFI hides the nuclear localization signal of MyoD family protein, leading to the accumulation of transcription factors in the cytoplasm. In mouse models, the suppression of MDFI expression promotes muscle regeneration after injury. MDFI has been also demonstrated to take part of a regulatory circuit in which the proto-oncogene FOS inhibits the expression of MDFI, which, in turn, promotes MyoD-induced differentiation. MyoD upregulates the miR-501-3p muscle-specific miRNA, which dampens FOS expression promoting myoblast differentiation in a negative regulatory feedback loop mechanism [83].

As expected, miRNAs not only participate in the promotion of myogenic differentiation, but they also inhibit this process suppressing MRFs expression. The overexpression of miR-124 is associated with low levels of myogenic transcription factors such as Myf5, MyoD, and myogenin [84]. A miR-221/222-MyoD-myomiRs regulatory pathway has been demonstrated to regulate the expression of miR-1, miR-133 miR-206, myomiRs in C2C12 cell models. [85]. In addition to MRFs, miRNAs may also inhibit factors belonging to the Mef2 family, which is composed of transcription factors that regulate the myogenic process in concert with MRFs. In vitro experiments demonstrated that miR-155 directly represses the expression of MEF2A isoform, contrasting cell differentiation [86].

Furthermore, selected miRNAs have been demonstrated to inhibit the myogenic differentiation acting on TGFβ and Wnt/β-catenin signaling. The myomiR miR-499 may inhibit TGFβ-receptor 1, promoting the proliferation of C2C12 cells [87], whereas miR-216a targets Smad7, a member of the inhibitory Smads family that can specifically inhibit TGFβ pathway and bovine primary muscle cells differentiation [88]. Inhibition of cell self-renewal progression of muscle precursors in the myogenic lineage has been attributed to miR-143-3p and miR-664-5p that downregulate Wnt5a, LRP5, Axin2, β-catenin and Wnt1 expression [89,90].

An additional mechanism by which satellite cells may contribute to muscle fiber adaptation influencing extracellular matrix related gene expression is the release of extracellular vesicles, such as exosomes [91,92]. Exosomes are small cell-secreted endocytic membrane-derived vesicles that contribute to cell-to-cell communication, transporting proteins, DNA, mRNA, miRNAs. During skeletal muscle modeling, miR-206 has been demonstrated to be secreted via exosomes by skeletal muscle myogenic progenitor cells and to target the ribosomal binding protein Rrbp1, which regulates the collagen expression in the extracellular matrix [60]. The relevant role of miRNAs delivered by extracellular vesicles in muscle fiber hypertrophy has been further demonstrated in mice in response to mechanical load. Not only mir-206 is enriched in vesicles derived by primary myogenic progenitor cells, but also several additional miRNAs, including mi-24, miR-149, mir-486, let-7e, miR-133 a/b and miR-320. All these miRNAs are known to regulate the process of extracellular matrix remodeling, reducing the expression of extracellular matrix-related genes such as matrix metalloproteinase 9 Mmp9 [93].

## 5. Role of miRNAs in Skeletal Muscle Aging and Sarcopenia

**Figure 2:**
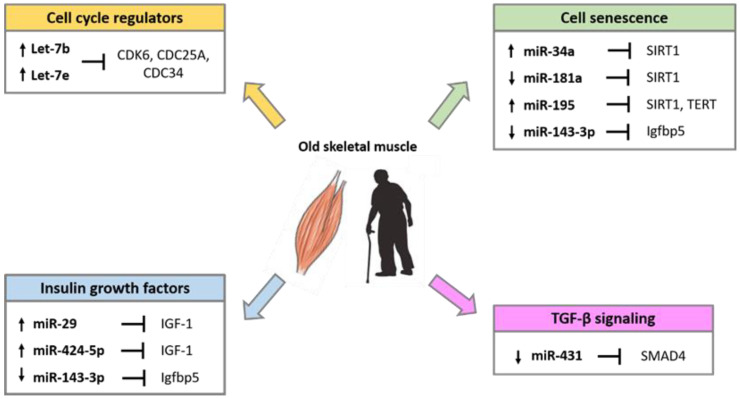
miRNAs involved in the regulation of age-related pathways in skeletal muscle.

Several studies have demonstrated that miRNAs are implicated in the alteration of skeletal muscle homeostasis and function during aging. Comparative studies in young and old muscle tissues of rodents, monkeys and humans showed differential expression of several miRNAs involved in the control of the myogenic processes [94,95,96]. The differentially expressed miRNA during aging, target factors that regulate cell cycle progression, the insulin-like growth factors (IGFs), factors involved in the process of cell senescence such as SIRT1 and telomerase reverse transcriptase (TERT), and factors of the transforming growth factor-β (TGF-β) cell signaling pathways (Figure 2).

miRNA expression profile analyses of skeletal muscle biopsy samples of healthy adults demonstrated that precursor of myomiRs miR-1, miR-133a, and miR-206 are upregulated in older subjects compared with younger ones [94]. This difference is not detected when analyzing the expression of mature miRNAs, suggesting that the maintenance of mature myomiRs might be useful to counteract muscle loss associated with aging. Higher expression of two miRNAs of the let-7 family, Let-7b and Let-7e, has been demonstrated in the elderly compared to young adults [97]. It has been proposed that the Let-7 miRNA family expression can reduce satellite cells proliferation in aged skeletal muscle by targeting regulators of cell cycle progression, such as CDK6, CDC25A and CDC34 [97].

Genome-wide miRNAs profile analyses in aging animal models confirm that miRNAs may be differentially expressed in old compared to young muscle [95,96]. In old muscle, a specific role has been attributed to miR-181a lower expression. During muscle differentiation, the miR-181 higher levels reduce the expression of the homeobox protein Hox-A11, a suppressor of MyoD, resulting in the upregulation of myogenin and other muscle marker proteins [98]. It has been proposed that the downregulation of miR-181a in old muscle tissue may lead to restriction of satellite cells proliferation, allowing a higher expression of activin receptor type IIA (ActRIIA), a member of the TGFβ receptor family, which suppresses cell proliferation via Smad2/3 phosphorylation [21]. As miR-181a controls the expression of well-known proinflammatory cytokines such as TNF-α, IL-6, IL-1β, and IL-8 [99], it is expected that miR-181a downregulation participates to inflammatory processes in the elderly. A more recent study showed that miR-181a and Sirtuin 1 (SIRT1) protein expression are inversely correlated in aged mice muscle [100]. Sirtuins have a crucial role in suppressing cellular senescence and extend longevity by interacting with several age-related signaling pathways such as insulin/insulin-like growth factor-1 (IGF-1), AMP-activated protein kinase, and forkhead box O [101]. It is reasonable to speculate that the downregulation of miR-181a may overcome SIRT1 repression and delay cellular senescence participating in the autophagic cell mechanisms. SIRT1 is an important regulator of autophagy and autophagy processes participate in maintaining muscle mass, neuromuscular communication, stem cell self-renewal and differentiation [102,103,104,105].

An additional miRNA, miR-195, has been recently demonstrated to decrease SIRT1 and TERT expression in old skeletal myoblasts [106]. Inhibition of miR-195 reversed the senescent into the juvenile phenotype, thus miR-195 may be a potential target for senescent cell fate reversion. Besides, miR-29 and miR-143-3p have been shown to be involved in the regulation of cellular senescence. miR-29 targets IGF-1 and the p85α regulatory subunit of PI3K and miR-29 overexpression is associated with high levels of the marker of cellular senescence, SA-βgal [107]. miR-143-3p expression is absent in the satellite cells and primary myoblasts of older mice and humans, whereas its target gene, insulin growth factor-binding protein 5 (Igfbp5), is present at high levels [108]. Age-related decrease of miR-143 expression may be a compensatory mechanism required to improve the myogenic process impaired by the concomitant increased levels of Igfbp5 that in turn induces cell senescence [109].

In mice, the ectopic expression of miR-431, which binds to a conserved site on SMAD4 3′ UTR, has been demonstrated to enhance old myoblasts differentiation and to restore the regeneration of the tissue, suggesting that miR-431 may play a key role in the maintenance of the myogenic capability of myoblasts with age [110].

miR-155, which is known to be involved in inflammatory processes, is upregulated in aged muscle satellite cells [109]. Notch1 normally binds to the promoter region of miR-155, participating in its suppression. In aged muscle, Notch1 expression is reduced, resulting in higher expression of miR-155. Both events, Notch 1 downregulation and miR155 overexpression therefore might contribute to inflammation status and muscle stem cell differentiation process [109].

Increased oxidative stress during aging can lead to muscle loss and function due to the activation of various catabolic pathways, including apoptosis. Recently, it has been observed that miR-434-3p is downregulated in the skeletal muscle of aged mice, and its expression negatively correlated with the levels of eukaryotic translation initiation factor 5A1 (eIF5A1), a factor demonstrated to induce apoptosis via the mitochondrial apoptotic pathway [111]. Low levels of miR-434-3p has also been detected in the serum of aged mice, indicating miR-434-3p as a potential biomarker of muscle atrophy related to age [10].

In sarcopenic people, several studies confirmed an accentuated increase in the expression level of miR-34a-5p, miR-449b-5p and miR-424-5p [11,112,113]. miR-34a targets cell senescence factors such as SIRT1 and the Endothelial Growth Factor A (VEGFA) and it should be considered a muscle aging key factor, whose expression is modulated in the cellular process leading to age-related skeletal muscle decline [114]. Moreover, in vitro and in vivo experiments show that miR-424-5p regulates the expression of transcripts that are involved in the synthesis of rRNA and its overexpression reduces the protein synthesis, a phenomenon that contributes to the loss of muscle mass in sarcopenia and aging, i.e., “anabolic resistance” [114]. Anabolic resistance may derive from a reduced muscle sensitivity to anabolic signaling as demonstrated for insulin, IGF-1 signaling, and inhibition of Akt/mToR by inflammatory signals. miR-424-5p was proposed to contribute to this component of anabolic resistance based on the evidence that IGF-1 is one of its most enriched targets [112]. Elevated expression of miR-424 detected in patients with muscle wasting correlates with a reduction of protein synthesis and loss of muscle mass [112]. The possibility to modulate miRNAs expression could provide potential therapeutic prospectives to enhance protein synthesis and reduce muscle loss in the elderly.

## 6. miRNAs Are Regulated by Exercise in Sarcopenia

Skeletal muscle exhibits an extraordinary plasticity dictated by a fine balance between anabolic and catabolic processes [2]. Muscle composition is influenced by internal and external stimuli (e.g., the intrinsic cellular aging process or environmental factors) that can accelerate or temporize the functional muscle impairment associated with aging and the sedentary behavior [15,16,115]. Indeed, aging is associated with a decrease in muscle protein synthesis [116], mitochondrial dysfunction, as well as a loss of fast glycolytic fibers and reduced blood perfusion [117,118,119]. Therefore, older people show a characteristic weakening, deterioration, and muscle loss, accompanied by microvascular impaired perfusion and an underlying state of inflammation [47,120]. This turnover is detrimental for healthy aging but is partially reversible with physical activity [121,122]. To date, lifestyle interventions and exercise training represent the primary, most effective, and non-pharmacological approach to prevent and treat sarcopenia-related decline.

Exercise is prescribed with different approaches, including customized type, frequency, intensity, and duration, to endorse positive outcomes. The most common and studied models of exercise can be distinguished in endurance and resistance: the latter is characterized by low intensity, long-duration aerobic activity, the former by higher intensities, short bouts with primary involvement of the anaerobic pathways. Endurance training is mainly known for its positive effects on the cardiovascular fitness (i.e., maximal oxygen consumption), but it also induces mitochondrial biogenesis, increases oxidative enzyme activity with a reduction in ROS generation, promotes muscle vascularization and regulates vascular tone [123,124]. The changes in muscle metabolism enhance the all-body aerobic fitness capacity (peak VO_2_ uptake) and attenuate the age-associated decline in the peripheral and central vasculature and cardiac functions [125,126]. Considering that the main feature of aging and sarcopenia is muscle atrophy, resistance exercise training is the most indicated, as it elicits the biosynthesis of contractile and structural proteins, resulting in muscle hypertrophy [127]. Specifically, resistance training significantly increases the number and cross-sectional area of type II fibers [123,124]. This improvement in muscle fibers results in a general improvement in functional capacity, muscular endurance, and quality of life [20].

miRNA expression may be adapted in response to exercise and altered miRNA profile may contribute to affect the plasticity of aged muscle [128,129]. Understanding the signaling pathways regulated by these miRNAs allows to shed light on the molecular mechanisms associated to improved muscle anatomy and function and may help establish specific protocols of training.

### 6.1. Exercise Modulates Muscle miRNAs Expression

Several studies have investigated the miRNAs changes to exercise. A comprehensive review of studies analyzing the miRNAs expression in human serum, plasma, whole blood, saliva, or muscle biopsy has been recently published [23]. To integrate more information and understand better the role of exercise on miRNAs expression, in this review, the pure long-duration moderate-intensity endurance exercise will be differentiated from the high-intensity endurance training. In these terms, a long-duration moderate-intensity intervention (>30 min @ ~70% VO_2max_) elicits mainly the aerobic processes and, if performed for 6 weeks, significantly increases mitochondrial capacity [130]. A wide gene expression analysis study indicates that 6 weeks of endurance training (4 days/week, 45min @ 70% VO_2max_) may reduce the expression of miR-1 and miR-133, together with miR-101 and miR-455 in young healthy adults [131]. On the other hand, medium-duration high-intensity endurance training utilizes intensities usually above the lactate threshold (~85% to 90% VO_2max_). The energy transformation is a combination of aerobic and anaerobic pathways, and the expression of myomiRs appears different. miR-1, miR-133a, miR-133b and miR-206 were downregulated at rest following 12 weeks of mixed-endurance training (2 days of continuous 60–150 min @ ~60% peak power output (PO_peak_), 2 days of interval 70–80 min @ 75–90% PO_peak_ and one day of maximal incremental test) and return to a high level of expression 14 days after cessation of regular training [132]. Additionally, increasing even more the exercise intensity, mixed endurance training (45–90 min @ 75% VO_2peak_) with high-intensity interval training (HIIT; 6 sets of 5 min @ 90–100% VO_2peak_) provided evidence that miR-1, miR-133a, miR-133b and miR-181a levels are increased, whereas miR-9, miR-23a, miR-23b and miR-31 are decreased [133]. Using reporter assays, miR-31 was reported to directly interact with HDAC4, which belongs to the MAPK pathway, and with Nuclear Respiratory Factor 1 (NRF1), which is involved in mitochondrial biogenesis and metabolism. To investigate if different types of exercise, even if all considered aerobic, have different outcomes on the myomiR expression, different endurance exercise modalities were explored on a group of young adults [134]. A single bout (90 min) of traditional non-weight-bearing endurance exercise on a cycle ergometer was compared to a walking trial with a loaded vest equivalent to the 30% of the individual body mass. MyomiR expression was different between the two modalities, even if the intensity was matched. The load carriage exercise diminished miR-1-3p, miR-206, miR-208a-5p, and miR-499 expression, while traditional endurance exercise on the cycle ergometer increased myomiR expression. In other words, the endurance exercise with a predominant anabolic part, resulted in diminished myomiR expression [134]. All the aforementioned studies investigated young adults, and further studies should explore if similar results occur in elderly people.

As mentioned before, resistance training is the more indicated and effective type of training for the older population. A limited number of studies have investigated the potential correlation between the decline of protein synthesis and anabolism of skeletal muscle during aging and the modulation in miRNA expression [94,135,136]. Representative examples of miRNAs specifically expressed in skeletal muscle modulate after exercise in young and elderly people are listed in Table 1.

In particular, Drummond et al. studied the impact of an acute resistance training session (8 sets of 10 repetitions @ 70% 1 maximal repetition (RM)), and for the first time determined that miR-1 skeletal muscle expression is downregulated in young men but not in older men, after stimulation of muscle protein synthesis [94]. As mentioned above, miR-1 targets many transcripts responsible for muscle growth and satellite cell function, such as the IGF-1 factor. Reduction of miR-1 expression may allow the activation of the mTORC1 pathway through IGF-1 signaling that, in turn, increases mRNA translational efficiency and muscle protein synthesis. The maintenance of miR-1 expression levels in the elderly results in reduced mTORC1-related intracellular events, which may explain the lack of anabolic response to the bout of resistance exercise in the elderly [137]. Evidence has been provided that the levels of thirteen miRNA species, including members of the miR-378, miR-30 and miR-128 families, were increased following 30 min of acute resistance exercise in adult healthy sedentary people [138]. Of particular interest is the role of miR-378a, which is located within intron 1 of the PPARGC1B gene, a coactivator of nuclear receptors and other transcription factors that regulate metabolic processes, including mitochondrial biogenesis and respiration, hepatic gluconeogenesis, and muscle fiber-type switching [139]. miR-378 isoforms have been implicated in the control of mitochondrial function and energy balance in mice, suggesting that miR-378 may participate in the adaptation of mitochondrial function and metabolic balance in the exercise [140]. Mouse models lacking miR-378-3p and -5p, but leaving the PPARGC1B gene intact, are resistant to diet-induced obesity, thus, the changes seen in miR-378 in muscle following exercise could also have effects on mitochondrial function and energy homeostasis [140].

HIIT, but not moderate–intensity continuous training has been shown to modulate the post-exercise expression of miR-133a, miR-378 and miR-486, all involved in skeletal muscle adaptation to resistance exercise. HIIT exercises are particularly effective in inducing anabolic responses increasing mTOR signaling and intermediates phosphorylation [141].

In agreement, Rivas et al. identified several miRNAs that are differentially expressed in young men, after an acute session of resistance exercise (6 sets of 10 reps @ 80% 1RM), whereas they did not show differences in older adults [135]. In vitro analysis demonstrated miR-126-3p, belonging to the differentially expressed miRNA, regulates the expression of Akt/forkhead box protein O1 (Foxo1), MyoD, MyF5 and factors of the IGF-1 signaling, suggesting its role in the control of muscle protein synthesis pathways related to aging. miRNA that regulates several members of the Akt-mTOR signaling, such as miR-99a-5p, miR-99b-5p, miR-100-5p, miR-149-3p, miR-196b-5p, and miR-199a have also been demonstrated to be differentially expressed in older men compared to the young, in response to acute resistance exercise (3 sets of 14 reps @ 60% 1RM); this confirmed the relevance of the Akt-mTOR signaling in aging-related muscle plasticity [136]. Since weakened Akt-mTOR signaling activation is associated with the progression of muscle wasting during aging, altered miRNA expression profile following acute anabolic stimulus may contribute to the progressive weakening of skeletal muscle in the elderly, contributing to the onset of sarcopenia. In a study that analyzed the effects of 5 months of resistance training (3 days/week, 3 sets of 10 reps @ 70% 1RM) on older adults, the expression levels of the myomiR miR-133b was decreased in muscle tissue [142].

From a study analyzing the hypertrophic response of young individuals that performed 12 weeks of intense resistance exercise training (RT), recruited subjects have been recognized and classified as “low responders” or “high responders” to the training [143]. Following the resistance exercise training, miR-451 levels resulted increased, whereas miR-26a, miR-29a and miR-378 expression were decreased only in the “low responders”. The reduced expression of miR-26a, miR-29a and miR-378 correlate to IGF-1 mRNA and increased only in the “high responders” group. The IGF-1 mRNA levels remained unchanged in the low-responder group post-training, suggesting that changing in the miRNA profile may be a compensatory mechanism for the lack of key target genes providing mRNA substrate to protein synthesis machinery. Thus, it has to be taken into account that exercise training always has an influence, but the level of intensity dictates the response and, consequently, the expression of the miRNAs.

### 6.2. Exercise Modulates Circulating miRNAs Expression

Although miRNAs are mainly detected in the intracellular environment, a great number of miRNAs, known as circulating miRNAs, have also been found in various biological fluids, such as blood, urine, and saliva, and are transported as signaling molecules to mediate cell-cell communications [144]. Determining the circulating miRNAs levels may be useful for the estimation of muscle function, recognizing them as non-invasive candidate biomarkers of sarcopenia or other skeletal muscles-associated disorders.

Differential expression of circulating miRNAs may represent a marker of response to gene expression regulation derived by environmental adaptation, including exercise. In plasma or serum, miRNAs are detectable in a remarkably stable form, encapsulated into the extracellular vesicles such as microvesicles, exosomes, apoptotic bodies or bound to RNA-binding proteins or high-density lipoproteins, making them resistant to RNase digestion. Circulating miRNA have been suggested to be mediators of gene expression, allowing cell-to-cell communication. Moreover, they have been suggested as potential non-invasive biomarkers to reveal muscle function and adaptation to exercise in the elderly [145,146,147]. In the frailty geriatric syndrome in addition to sarcopenia, mitochondrial dysfunction, oxidative stress, aging-related loss of anabolic hormones, decreased peripheral perfusion and chronic inflammation (inflammaging) are reported that could be associated with altered presence of circulating miRNAs [148,149,150,151].

A recent study observed that miR-21 levels were increased in frailty condition, whereas miR-146a were downregulated [152]. A decrease of miR-146a expression in frailty has been shown to induce NF-κB-dependent inflammation. Moreover, an increase in miR-483 level, which regulates the synthesis of the anti-aging hormone, melatonin, has been reported in frail patients [152].

The altered miRNA profile has also been demonstrated by comparing the expression of exosome-derived miRNA isolated from the plasma of young adults, robust, and frail elderly people [153]. The expression of a group of eight miRNAs (miR-10a-3p, miR-92a-3p, miR-185–3p, miR-194–5p, miR-326, miR-532–5p, miR-576–5p, and miR-760) was found altered with higher expression in frail older individuals. These miRNAs are predicted to be implicated in aging pathways such as the insulin signaling pathway, the AMPK signaling pathway, and the FoxO signaling pathway.

Altered expression of circulating miRNAs have been demonstrated in response to acute and chronic exercise, and have been extensively described in a recent review [154].

The lack of anabolic response to resistance exercise in the elderly can be found in the altered expression of circulating miRNA profile [140]. Nine circulating miRNAs (miR-19b-3p, miR-195-5p, miR-19a-3p, miR-106-5p, miR-20a-5p, miR-17-5p, miR-143-3p, miR-26b-5p, miR-93-5p) show a greater expression in younger compared to older subjects after exercise. Bioinformatics analyses of the potential targets of these miRNAs, showed the absence of factors involved in anabolic signaling, IGF-1, and mTOR signaling in older subjects, suggesting that a downregulation of these circulating miRNAs is indicative of “anabolic resistance” in elderly [155]. In addition to exercise, energy restriction influences the expression of circulating miRNAs that are inversely associated with whole-body protein synthesis [156]. Circulating miRNAs modulated by the exercise in the elderly are listed in Table 2.

A recent study has demonstrated that comparing two groups, one of older adults with at least 5 consecutive years of endurance-trained and one of sedentary males (≤1 day/weeks exercise), regular exercise significantly increased the expression of three miRs present in the exosomes, named exomiRS (miR-486-5p, miR-215-5p, miR-941) and decreased the expression of one exomiR (miR-151b) [160]. Acute exercise altered circulating exomiR expression in both trained groups. Pathway analysis prediction and validation experiments reported that the majority of exercises regulated exomiRs target transcripts of genes that are related to IGF-1 signaling [160].

Modified expression of circulating miRNAs has also been shown to correlate with exercise and diet weight loss intervention. Circulating miRNA-221 and -223 were found to be modulated following exercise and diet [159]. Studies on male marathon runners have demonstrated that prolonged aerobic exercises may change the expression of circulating miRNAs, such as miR-126 and miR-133, which may be potentially used as biomarkers of acute responses to physiological stress [157,158].

Further studies that will analyze the expression of circulating miRNAs in aging and in the adaptation to a healthy lifestyle, such as dietary choices and exercise may provide knowledge on the molecular mechanisms that sustain skeletal muscle structure and functionality.

## 7. Conclusions

Sarcopenia-related muscle dysfunction significantly affects life quality of elderly people. Skeletal muscle development, homeostasis, and metabolism during aging undergo changes in terms of gene expression regulation in which miRNAs have been demonstrated to play a crucial role. With aging, the altered expression profile of miRNAs that regulate the central processes of muscle cell biology contributes to the loss of muscle mass. Further studies on the molecular mechanisms of regulation of miRNAs during the multiple steps of skeletal myogenesis, as well as in sarcopenic/atrophy conditions will shed light to our knowledge on the functionality of skeletal muscle and on the identification of potential targets for therapies. Detection of circulating miRNAs in serum and plasma that can mirror skeletal muscle status is a potential opportunity to identify non-invasive biomarkers to improve clinical diagnosis and prognosis of sarcopenia.

## Figures and Tables

**Table 1:** Expression of skeletal muscle miRNAs after exercise reviewed in the text.

Subjects	Type of Exercise	Duration ^a^	MyomiRs	Other miRNAs	References
Older man(70 yr)	Resistance acute exercise bout	3/6 hrs	↓miR-1		[94]
30–35 yr old healthy, trained men	Endurance acute exercise bout	1 hr	↑miR-1, miR-133a/b, miR-206		[132]
	Endurance training	12 wks	↓miR-1, miR-133a/b, miR-206		
18–30 yr old men (low responders)	Resistance exercise training	12 wks		↑miR-451, ↓miR-26a, miR-29a, miR-378	[143]
24 yr old young sedentary healthy men	Endurance training	6 wks	↓miR-1, miR-133a	↓miR-101, miR-455	[131]
Males 23 yr old	Endurance acute exercise bout	3 hrs	↑miR-1, miR-133a/b, miR-206	↑miR-181a, ↓miR-9, miR-23a/b, miR-31	[133]
Healthy young (18–30 yr old) and older (60–75 yr) men	Resistance acute exercise bout	2 hrs	**Young**		[136]
			↑miR-486-3p,	↓miR-149-3p, miR-520g-3p, miR-99b-5p, miR-100-5p	
			**Old**		
			↑miR-499a-5p	↑ miR-99a-5p, ↓miR-186-5p, miR-196b, miR-335-5p, miR-628-5p, miR-489-5p	
Young, healthy (22 yr) and old (74 yr) men	Resistance acute exercise bout	6 hrs	**Young**		[135]
			↓miR-133a, miR-133b	↓miR-23b-3p, miR-24-3p, miR-26a-5p, miR-26b-5p, miR-27a-3p, miR-27b-3p, miR-29c-3p, miR-30a-5p, miR-30d-5p, miR-95-3p, miR-126-3p, miR-140-3p, miR-181a-5p	
Older men and women (65–80 yr)	Resistance exercise training	5 mos	↓miR-133b		[142]
Sedentary Healthy men and women (31–35 yr old)	Resistance acute exercise bout	30 min		↑miR-10a-5p, miR-30a-5p, miR-30d-5p, miR-22-3p, miR-128, miR-378a-3p, miR-378f, miR-378a-5p, miR-378g, miR-378i, miR-422a, miR-532-5p	[138]
Healthy males (23–31 yr old)	HIIT or MICT ^b^ + RE	1/3 hrs	↓miR-486, miR-133a	↓miR-378	[141]
25 adults (18–27 yr old)	Endurance single 90 min exercise bout (Load carriage or cycle ergometry)	Immediately/3 hrs	↓miR-1-3p, miR-206, miR-208a-5, miR-499		[134]

^a^ hrs, hours; mos, months; min, minutes; yr, year; wks, weeks. ^b^ High-intensity interval training (HIIT) or moderate-intensity continuous training (MICT); Resistance Exercise (RE).

**Table 2:** Circulating miRNAs differentially expressed in elderly compared to healthy young after exercise.

Subjects	Type of Exercise	Duration ^a^	c-miRNAs ^b^		Reference
**Male marathon runners (51.6–62 yr old)**	Endurance acute exercise bout	30 min/4 hrs	**Immediately post-exercise:** **↑**miR-126, miR-133		[157]
**Male marathon runners (50.4–53.2 yr old)**	Endurance acute exercise bout	Immediately post/up to 24 hrs	**Immediately post exercise:** **↑**miR-1, miR-126, miR-133a, miR-134, miR-146a, miR-208a, miR-499-5p		[158]
**Healthy, inactive, young (21–23 yr) and old (72–76 yr)**	Resistance acute exercise bout	6 hrs	**Young** **↑**miR-19b-3p, miR-195-5p, miR-19a-3p, miR-106-5p, miR-20a-5p, miR-17-5p, miR-143-3p, miR-26b-5p, miR-93-5p		[155]
**Low (40.6–52 yr) and high (42–54.4 yr) responding men and women to a weight loss intervention**	Resistance exercise training and diet intervention	16 wks	**Both groups:** **↑**221-3p, 223-3p **Only Low responder:** **↑**miR-140		[159]
**Older trained and older sedentary men (>65 yr old)**	Endurance acute exercise bout	40 min	**Trained group**		[160]
			**Immediately post exercise** **↑**miR-383-5p, miR-339-5p, miR-874-3p **↓**miR-206, miR-486-5p, miR-148a-3p, let-7b-5p	**3 hrs post exercise↑**miR-34b-3p, miR-129-2-3p, miR-138-1-3p, miR-671-3p, miR-885-5p **↓**miR-486-5p, miR-629-5p, miR-16-2-3p	
			**Sedentary group**		
			**Immediately post exercise** **↑** miR-505-3p, miR-29b-3p, miR-203a-3p, miR-384, miR-451a, miR-223-3p, miR-218-5p, miR-495-3p	**3 hrs post exercise** **↓**miR-4433b-3p, miR-378c, miR-151b, miR-151a-5p, hsa- miR-151b	

^a^ hrs, hours; mos, months; min, minutes; yr, year; wks, weeks. ^b^ c-miRNAs, irculating miRNA.
